# Supplementary material for: Pan-Cancer Analysis Identifies MNX1 and Associated Antisense Transcripts as Biomarkers for Cancer
Source: Cells. 2022 Nov 11;11(22):3577. doi: 10.3390/cells11223577 (PMC9688723; doi:10.3390/cells11223577)
Supplement: Supplementary file 1 [file cells-11-03577-s001.zip › cells-1929373-supplementary.pdf]

# Supplementary Materials

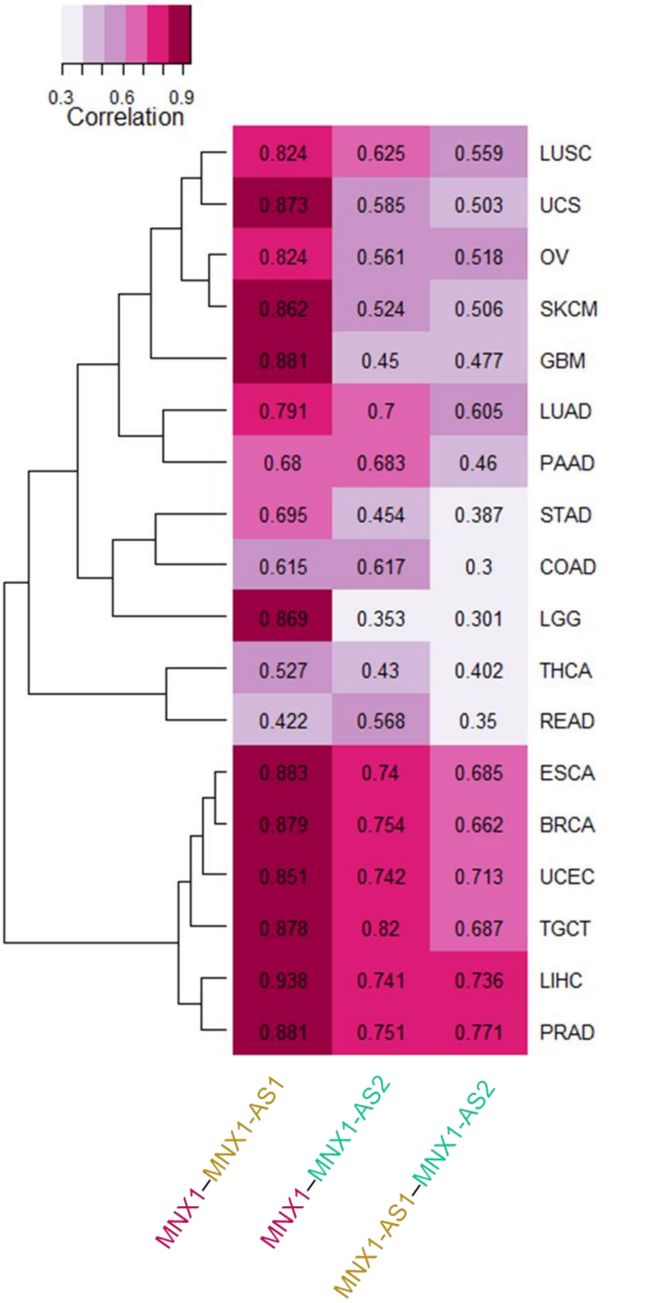


**Supplementary Figure S1.** Hierarchical clustering on Pearson correlation values of expression between MNX1-MNX1-AS1, MNX1-MNX1-AS2, and MNX1-AS1-MNX1-AS2 in each cancer type analysed.

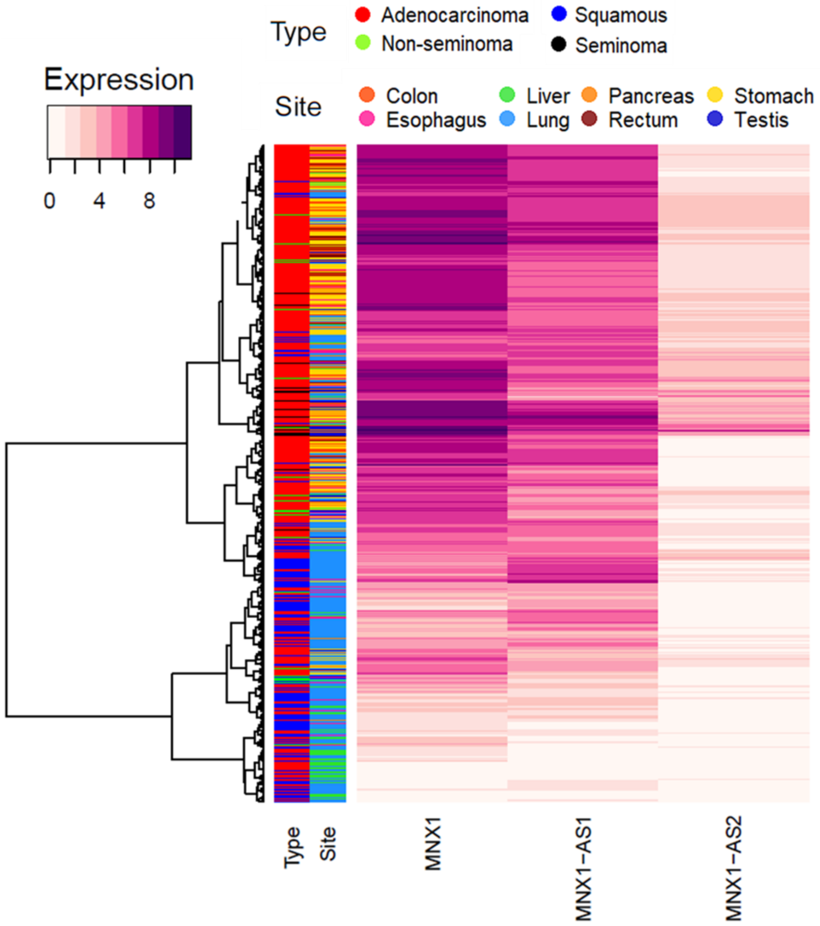


**Supplementary Figure S2.** Complete hierarchical clustering of MNX1, MNX1-AS1 and MNX1-AS2 expression levels, with cancer type and site information, complementary to the binary clustering in Figure 6.

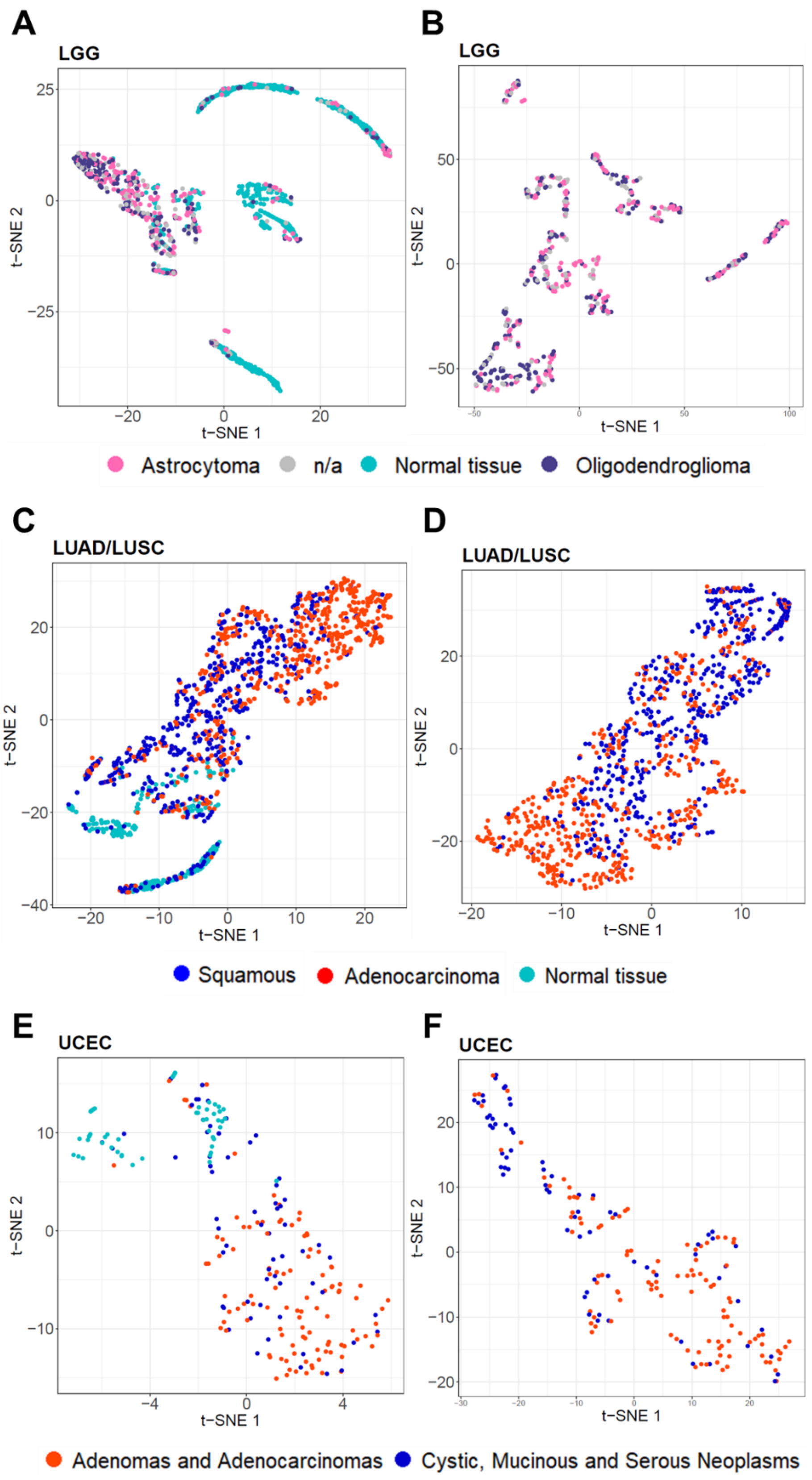


**Supplementary Figure S3.** Site-specific t-SNE plots for discriminating disease subtypes based on MNX1, MNX1-AS1, and MNX1-AS2 expressions. **(a-b)** Clustering of astrocytomas and oligodendrogliomas in LGG, by comparison with normal brain tissue and tumour-only analysis. **(c-d)** t-SNE analysis of LUAD against LUSC samples and normal lung tissue. **(e-f)** Differentiation of adenocarcinomas and cystic, mucinous and serous cancers in UCEC, and normal uterine tissue.

**Supplementary Table S1.** Statistically significant correlations between expression levels of *MNX1*, *MNX1-AS1*, and *MNX1-AS2* and clinicopathological features.

| | | *MNX1* | | | *MNX1-AS1* | | | *MNX1-AS2* | | |
|---|---|---|---|---|---|---|---|---|---|---|
| | | **high** | **low** | **p** | **high** | **low** | **p** | **high** | **low** | **p** |
| **BRCA** | **Age** | | | | | | | | | |
| | < 50 years | 149 | 179 | 0.027 | 154 | 174 | 6.25E-04 | | | ns |
| | ≥ 50 years | 402 | 361 | | 444 | 319 | | | | |
| **COAD** | **Metastasis (M)** | | | | | | | | | |
| | Yes (M1) | 30 | 10 | 0.002 | 29 | 11 | 0.046 | | | ns |
| | No (M0) | 93 | 100 | | 107 | 86 | | | | |
| | **Stage** | | | | | | | | | |
| | Early (I, II) | 70 | 84 | 0.035 | | | ns | 67 | 87 | 0.043 |
| | Late (III, IV) | 71 | 51 | | | | | 68 | 54 | |
| | **Disease type** | | | | | | | | | |
| | Adenoma | 138 | 110 | 0.006 | 154 | 94 | 0.0001 | | | ns |
| | Cystic, mucinous and serous | 13 | 27 | | 12 | 28 | | | | |
| **ESCA** | **Lymph nodes (N)** | | | | | | | | | |
| | Yes | 56 | 32 | 0.003 | 54 | 34 | 0.032 | 48 | 40 | 1.35E-02 |
| | No | 30 | 44 | | 33 | 41 | | 26 | 48 | |
| | **Disease type** | | | | | | | | | |
| | Adenocarcinoma | 82 | 7 | 2.20E-16 | 73 | 16 | 1.93E-11 | 65 | 24 | 1.36E-11 |
| | Squamous | 22 | 70 | | 30 | 62 | | 21 | 71 | |
| | **Grade (histologic)** | | | | | | | | | |
| | Low (G1, G2) | 42 | 50 | 1.40E-02 | 42 | 50 | 6.71E-04 | 33 | 59 | 2.78E-02 |
| | High (G3, G4) | 33 | 16 | | 37 | 12 | | 27 | 22 | |
| **GBM** | **Gender** | | | | | | | | | |
| | Male | | | ns | 52 | 46 | 0.0345 | | | ns |
| | Female | | | | 19 | 35 | | | | |
| **LGG** | **Age** | | | | | | | | | |
| | < 50 years | 145 | 198 | 0.004 | 135 | 208 | 0.005 | | | ns |
| | ≥ 50 years | 87 | 68 | | 82 | 73 | | | | |

| | | | | | | | | | | |
|---|---|---|---|---|---|---|---|---|---|---|
| | **Disease type** | | | | | | | | | |
| | Astrocytoma | | | ns | 72 | 121 | 0.001 | | | ns |
| | Oligodendroglioma | | | | 101 | 87 | | | | |
| | **Grade (histologic)** | | | | | | | | | |
| | Low (G1, G2) | 102 | 145 | 0.032 | | | ns | 100 | 147 | 0.050 |
| | High (G3, G4) | 132 | 128 | | | | | 127 | 133 | |
| **LIHC** | **Age** | | | | | | | | | |
| | < 50 years | 29 | 42 | 0.027 | 26 | 45 | 0.037 | | | ns |
| | ≥ 50 years | 80 | 211 | | 71 | 220 | | | | |
| | **Gender** | | | | | | | | | |
| | Male | 54 | 195 | 3.51E-08 | 47 | 202 | 1.35E-07 | 37 | 212 | 1.82E-07 |
| | Female | 60 | 60 | | 54 | 66 | | 47 | 73 | |
| | **Grade (histologic)** | | | | | | | | | |
| | Low (G1, G2) | 62 | 170 | 0.018 | 52 | 180 | 0.004 | 43 | 189 | 0.010 |
| | High (G3, G4) | 51 | 81 | | 48 | 84 | | 40 | 92 | |
| | **Lymph nodes (N)** | | | | | | | | | |
| | Yes (N1, N2, N3) | 4 | 0 | 0.002 | 4 | 0 | 0.001 | | | ns |
| | No (N0) | 75 | 175 | | 68 | 182 | | | | |
| | **Stage** | | | | | | | | | |
| | Early (I, II) | 70 | 185 | 0.028 | | | ns | 51 | 204 | 0.015 |
| | Late (III, IV) | 33 | 49 | | | | | 27 | 55 | |
| **LUSC** | **Lymph nodes (N)** | | | | | | | | | |
| | Yes (N1, N2, N3) | 104 | 86 | 0.013 | 116 | 74 | 1.37E-04 | | | ns |
| | No (N0) | 153 | 198 | | 154 | 197 | | | | |
| **Lung** | **Disease type** | | | | | | | | | |
| | LUAD (adenocarcinoma) | 383 | 191 | 2.2E-16 | 382 | 192 | 7.12E-14 | 338 | 236 | 2.2E-16 |
| | LUSC (squamous) | 187 | 361 | | 246 | 305 | | 139 | 409 | |
| **OV** | **Stage** | | | | | | | | | |
| | Early (I, II) | 17 | 8 | 0.008 | | | ns | | | ns |
| | Late (III, IV) | 161 | 231 | | | | | | | |
| **PAAD** | **Tumor size (T)** | | | | | | | | | |
| | < 5 cm | 22 | 9 | 0.010 | | | ns | | | ns |

| | | | | | | | | | | |
|---|---|---|---|---|---|---|---|---|---|---|
| | ≥ 5 cm | 66 | 79 | | | | | | | |
| | **Grade (histologic)** | | | | | | | | | |
| | Low (G1, G2) | | | ns | 57 | 69 | 0.006 | | | ns |
| | High (G3, G4) | | | | 34 | 16 | | | | |
| **PRAD** | **Age** | | | | | | | | | |
| | < 50 years | 10 | 24 | 0.002 | 9 | 25 | 4.47E-04 | 11 | 23 | 0.023 |
| | ≥ 50 years | 261 | 200 | | 265 | 196 | | 242 | 219 | |
| | **Lymph nodes (N)** | | | | | | | | | |
| | Yes (N1, N2, N3) | 56 | 23 | 0.003 | 57 | 22 | 0.004 | 50 | 29 | 0.043 |
| | No (N0) | 180 | 163 | | 187 | 156 | | 174 | 169 | |
| | **Tumor size (T)** | | | | | | | | | |
| | < 5 cm | 78 | 109 | 7.65E-06 | 75 | 112 | 6.48E-08 | 77 | 110 | 4.62E-04 |
| | ≥ 5 cm | 188 | 113 | | 196 | 105 | | 173 | 128 | |
| **STAD** | **Gender** | | | | | | | | | |
| | Male | 160 | 108 | 4.19E-02 | | | ns | | | ns |
| | Female | 72 | 74 | | | | | | | |
| | **Lymph nodes (N)** | | | | | | | | | |
| | Yes (N1, N2, N3) | 143 | 128 | 0.005 | | | ns | | | ns |
| | No (N0) | 84 | 40 | | | | | | | |
| **TGCT** | **Lymph nodes (N)** | | | | | | | | | |
| | Yes (N1, N2, N3) | | | ns | | | ns | 8 | 22 | 0.041 |
| | No (N0) | | | | | | | 39 | 42 | |
| | **Disease type** | | | | | | | | | |
| | Seminoma | 53 | 13 | 4.29E-11 | 48 | 18 | 4.48E-07 | 48 | 18 | 4.68E-11 |
| | Non-seminoma | 17 | 54 | | 21 | 50 | | 12 | 59 | |
| **UCEC** | **Disease type** | | | | | | | | | |
| | Adenoma | 78 | 28 | 1.60E-06 | 75 | 32 | 3.33E-07 | 64 | 43 | 4.19E-05 |
| | Cystic,mucinous and serous | 27 | 46 | | 23 | 50 | | 21 | 52 | |

**Supplementary Table S2.** P values of survival analysis by Kaplan-Meier analysis based on high and low individual expressions of MNX1, MNX1-AS1 and MNX1-AS2, as well as combinatorial ("COMB"). P values lower than 0.05 are highlighted.

| | OS | | | | DFI | | | PFI | | |
|---|---|---|---|---|---|---|---|---|---|---|
| | MNX1 | MNX1-AS1 | MNX1-AS2 | COMB | MNX1 | MNX1-AS1 | MNX1-AS2 | MNX1 | MNX1-AS1 | MNX1-AS2 |
| **BRCA** | 0.8 | 0.24 | 0.74 | 0.45 | 0.75 | 0.19 | 0.92 | 0.61 | 0.21 | 0.73 |
| **COAD** | 0.033 | 0.046 | 0.029 | 0.018 | 0.22 | 0.8 | 0.11 | 0.097 | 0.17 | 0.033 |
| **ESCA** | 0.68 | 0.66 | 0.84 | < 0.001 | 0.25 | 0.76 | 0.08 | 0.62 | 0.22 | 0.85 |
| **GBM** | 0.59 | 0.59 | 0.16 | 0.079 | N/A | N/A | N/A | 0.88 | 0.51 | 0.055 |
| **LGG** | 0.28 | 0.39 | 0.089 | < 0.001 | 0.63 | 0.84 | 0.40 | 0.77 | 0.84 | 0.028 |
| **LIHC** | 0.79 | 0.77 | 0.6 | 0.45 | 0.32 | 0.32 | 0.41 | 0.26 | 0.68 | 1 |
| **LUAD** | 0.41 | 0.082 | 0.98 | 0.73 | 0.024 | 0.22 | 0.073 | 0.58 | 0.097 | 0.85 |
| **LUSC** | 0.79 | 0.8 | 0.31 | 0.53 | 0.074 | 0.67 | 0.94 | 0.017 | 0.81 | 0.68 |
| **OV** | 0.78 | 0.032 | 0.15 | 0.087 | 0.71 | 0.0066 | 0.48 | 0.72 | 0.16 | 0.29 |
| **PAAD** | 0.064 | 0.43 | 0.8 | 0.13 | 0.29 | 1 | 0.092 | 0.22 | 0.99 | 0.79 |
| **PRAD** | 0.22 | 0.21 | 0.87 | 0.65 | 0.15 | 0.19 | 0.85 | 0.066 | 0.19 | 0.57 |
| **READ** | 0.43 | 0.43 | 0.21 | 0.81 | 0.99 | 0.82 | 0.14 | 0.71 | 0.16 | 0.41 |
| **SKCM** | 0.77 | 0.87 | 0.074 | 0.49 | N/A | N/A | N/A | 0.39 | 0.35 | 0.17 |
| **STAD** | 0.022 | 0.47 | 0.59 | 0.15 | 0.16 | 0.41 | 0.29 | 0.024 | 0.44 | 0.76 |
| **TGCT** | 0.48 | 0.47 | 0.63 | 0.99 | 0.61 | 0.35 | 0.88 | 0.74 | 0.76 | 0.74 |
| **THCA** | 0.26 | 0.33 | 0.11 | 0.28 | 0.085 | 0.056 | 0.87 | 0.14 | 0.057 | 0.75 |
| **UCEC** | 0.62 | 0.48 | 0.53 | 0.054 | 0.64 | 0.4 | 0.61 | 0.63 | 0.95 | 0.44 |
| **UCS** | 0.33 | 0.88 | 0.95 | 0.46 | 0.4 | 0.85 | 0.17 | 0.25 | 0.59 | 0.89 |

**Supplementary Table S3.** Evaluation of the sample data bias impact on the biological insights. Expression data from cancer (indicated by the tumour abbreviation) and normal samples (indicated by the tissue name) have been randomly split into two groups and the difference in the average expression in each group was evaluated using Wilcoxon test.

| Gene | P Value | Method | Site |
|---|---|---|---|
| MNX1 | 0.24 | Wilcoxon | Ovary |
| MNX1 | 0.17 | Wilcoxon | Uterus |
| MNX1 | 0.98 | Wilcoxon | Breast |
| MNX1 | 0.5 | Wilcoxon | Brain |
| MNX1 | 0.19 | Wilcoxon | Thyroid Gland |
| MNX1 | 0.36 | Wilcoxon | Lung |
| MNX1 | 0.17 | Wilcoxon | Pancreas |
| MNX1 | 0.51 | Wilcoxon | Esophagus |
| MNX1 | 0.85 | Wilcoxon | Stomach |
| MNX1 | 0.94 | Wilcoxon | Skin |
| MNX1 | 0.93 | Wilcoxon | Colon |
| MNX1 | 1 | Wilcoxon | Prostate |
| MNX1 | 0.77 | Wilcoxon | Testis |
| MNX1 | 0.69 | Wilcoxon | Liver |
| MNX1 | 0.48 | Wilcoxon | Kidney |
| MNX1 | 0.51 | Wilcoxon | GBM |
| MNX1 | 0.83 | Wilcoxon | OV |
| MNX1 | 0.74 | Wilcoxon | LUAD |
| MNX1 | 0.72 | Wilcoxon | LUSC |
| MNX1 | 0.63 | Wilcoxon | PRAD |
| MNX1 | 0.61 | Wilcoxon | UCEC |
| MNX1 | 0.9 | Wilcoxon | TGCT |
| MNX1 | 0.77 | Wilcoxon | ESCA |
| MNX1 | 0.61 | Wilcoxon | PAAD |
| MNX1 | 0.22 | Wilcoxon | KIRP |
| MNX1 | 0.47 | Wilcoxon | LIHC |
| MNX1 | 0.2 | Wilcoxon | BRCA |
| MNX1 | 0.29 | Wilcoxon | COAD |
| MNX1 | 0.53 | Wilcoxon | STAD |
| MNX1 | 0.96 | Wilcoxon | KIRC |
| MNX1 | 0.28 | Wilcoxon | THCA |
| MNX1 | 0.51 | Wilcoxon | READ |
| MNX1 | 0.38 | Wilcoxon | SKCM |
| MNX1 | 0.89 | Wilcoxon | LGG |
| MNX1 | 0.86 | Wilcoxon | KICH |
| MNX1 | 0.082 | Wilcoxon | UCS |
| MNX1.AS1 | 0.77 | Wilcoxon | Ovary |
| MNX1.AS1 | 0.96 | Wilcoxon | Uterus |
| MNX1.AS1 | 0.47 | Wilcoxon | Breast |
| MNX1.AS1 | 0.056 | Wilcoxon | Brain |
| MNX1.AS1 | 0.57 | Wilcoxon | Thyroid Gland |
| MNX1.AS1 | 0.99 | Wilcoxon | Lung |

| | | | |
|---|---|---|---|
| **MNX1.AS1** | 0.18 | Wilcoxon | Pancreas |
| **MNX1.AS1** | 0.37 | Wilcoxon | Esophagus |
| **MNX1.AS1** | 0.51 | Wilcoxon | Stomach |
| **MNX1.AS1** | 0.3 | Wilcoxon | Skin |
| **MNX1.AS1** | 0.63 | Wilcoxon | Colon |
| **MNX1.AS1** | 0.6 | Wilcoxon | Prostate |
| **MNX1.AS1** | 0.32 | Wilcoxon | Testis |
| **MNX1.AS1** | 0.29 | Wilcoxon | Liver |
| **MNX1.AS1** | 0.16 | Wilcoxon | Kidney |
| **MNX1.AS1** | 0.11 | Wilcoxon | GBM |
| **MNX1.AS1** | 0.7 | Wilcoxon | OV |
| **MNX1.AS1** | 0.39 | Wilcoxon | LUAD |
| **MNX1.AS1** | 0.54 | Wilcoxon | LUSC |
| **MNX1.AS1** | 0.46 | Wilcoxon | PRAD |
| **MNX1.AS1** | 0.74 | Wilcoxon | UCEC |
| **MNX1.AS1** | 0.71 | Wilcoxon | TGCT |
| **MNX1.AS1** | 0.95 | Wilcoxon | ESCA |
| **MNX1.AS1** | 0.36 | Wilcoxon | PAAD |
| **MNX1.AS1** | 0.77 | Wilcoxon | KIRP |
| **MNX1.AS1** | 0.48 | Wilcoxon | LIHC |
| **MNX1.AS1** | 0.28 | Wilcoxon | BRCA |
| **MNX1.AS1** | 0.44 | Wilcoxon | COAD |
| **MNX1.AS1** | 0.19 | Wilcoxon | STAD |
| **MNX1.AS1** | 0.28 | Wilcoxon | KIRC |
| **MNX1.AS1** | 0.24 | Wilcoxon | THCA |
| **MNX1.AS1** | 0.29 | Wilcoxon | READ |
| **MNX1.AS1** | 0.38 | Wilcoxon | SKCM |
| **MNX1.AS1** | 0.74 | Wilcoxon | LGG |
| **MNX1.AS1** | 0.9 | Wilcoxon | KICH |
| **MNX1.AS1** | 0.0075 | Wilcoxon | UCS |
| **MNX1.AS2** | 0.67 | Wilcoxon | Ovary |
| **MNX1.AS2** | 0.74 | Wilcoxon | Uterus |
| **MNX1.AS2** | 0.51 | Wilcoxon | Breast |
| **MNX1.AS2** | 0.24 | Wilcoxon | Brain |
| **MNX1.AS2** | 0.65 | Wilcoxon | Thyroid Gland |
| **MNX1.AS2** | 0.85 | Wilcoxon | Lung |
| **MNX1.AS2** | 0.62 | Wilcoxon | Pancreas |
| **MNX1.AS2** | 0.47 | Wilcoxon | Esophagus |
| **MNX1.AS2** | 0.93 | Wilcoxon | Stomach |
| **MNX1.AS2** | 0.44 | Wilcoxon | Skin |
| **MNX1.AS2** | 0.98 | Wilcoxon | Colon |
| **MNX1.AS2** | 0.58 | Wilcoxon | Prostate |
| **MNX1.AS2** | 0.79 | Wilcoxon | Testis |
| **MNX1.AS2** | 0.79 | Wilcoxon | Liver |
| **MNX1.AS2** | 0.81 | Wilcoxon | Kidney |
| **MNX1.AS2** | 0.33 | Wilcoxon | GBM |

| | | | |
|---|---|---|---|
| **MNX1.AS2** | 0.77 | Wilcoxon | OV |
| **MNX1.AS2** | 0.55 | Wilcoxon | LUAD |
| **MNX1.AS2** | 0.11 | Wilcoxon | LUSC |
| **MNX1.AS2** | 0.09 | Wilcoxon | PRAD |
| **MNX1.AS2** | 0.83 | Wilcoxon | UCEC |
| **MNX1.AS2** | 0.93 | Wilcoxon | TGCT |
| **MNX1.AS2** | 0.78 | Wilcoxon | ESCA |
| **MNX1.AS2** | 0.28 | Wilcoxon | PAAD |
| **MNX1.AS2** | 0.38 | Wilcoxon | KIRP |
| **MNX1.AS2** | 0.35 | Wilcoxon | LIHC |
| **MNX1.AS2** | 0.36 | Wilcoxon | BRCA |
| **MNX1.AS2** | 0.58 | Wilcoxon | COAD |
| **MNX1.AS2** | 0.91 | Wilcoxon | STAD |
| **MNX1.AS2** | 0.91 | Wilcoxon | KIRC |
| **MNX1.AS2** | 0.78 | Wilcoxon | THCA |
| **MNX1.AS2** | 0.28 | Wilcoxon | READ |
| **MNX1.AS2** | 0.91 | Wilcoxon | SKCM |
| **MNX1.AS2** | 0.48 | Wilcoxon | LGG |
| **MNX1.AS2** | 0.86 | Wilcoxon | KICH |
| **MNX1.AS2** | 0.56 | Wilcoxon | UCS |
